# Editorial: Methods to modulate sleep with neurotechnology, devices, or wearables

**DOI:** 10.3389/fnins.2026.1792469

**Published:** 2026-02-25

**Authors:** Matthew J. Reid, William G. Coon, Michael T. Smith

**Affiliations:** 1 School of Medicine, Johns Hopkins University, Baltimore, MD, United States; 2 Johns Hopkins Applied Physics Laboratory, Laurel, MD, United States; 3 Whiting School of Engineering, Johns Hopkins University, Baltimore, MD, United States

**Keywords:** EEG, polysomnography (PSG), sleep, sleep disturbance, wearables

Sleep is fundamental to physical health and wellbeing, yet chronic sleep disturbances remain pervasive across clinical and healthy populations (Grandner, 2022). However, traditional tools for sleep assessment (i.e., polysomnography), first-line interventions such as cognitive behavioral therapy for insomnia (CBT-I), and pharmacological sleep medications face persistent challenges related to accessibility, scalability, adherence, tolerance, and efficacy (Sateia et al., 2017; Baron and Hooker, 2017; Koffel et al., 2018; Manber et al., 2023). These challenges complicate the existing treatment landscape and create an unmet clinical need—one that modern technologies are increasingly positioned to address by complementing traditional treatment modalities (Perez-Pozuelo et al., 2020).

This Research Topic, *Methods to Modulate Sleep with Neurotechnology, Devices, or Wearables*, brings together novel methodological contributions and applied interventions that collectively illustrate the emerging landscape of data-driven, real-time approaches to improving sleep. The articles span mechanistic reviews, human laboratory studies, randomized controlled trials, and computational innovations, together highlighting the promise and challenges of next-generation sleep-modulating technologies.

A central conceptual anchor for this Topic is the comprehensive review by Coon et al., which synthesizes 15 years of progress in closed-loop neurostimulation of sleep. This review details how three key oscillatory targets—slow oscillations, sleep spindles, and hippocampal ripples—govern memory consolidation and are reliably modulated by phase-locked auditory, electrical, magnetic, thermal, or ultrasound-based stimulation. This review also outlines persistent engineering challenges: the importance of precise timing of stimuli, refractory periods that limit the number of effective stimuli, and the need for individualized, state-aware calibration to prevent arousals. Crucially, the authors propose a roadmap for a modular, open-source ecosystem for sleep neurotechnology that integrates real-time decoding, flexible effectors, and wearable or ambient sensors. This systems-level perspective provides the broader context into which the experimental studies of this Research Topic naturally fit.

Several contributions on this topic build directly on these principles by testing real-world devices designed to improve sleep through closed-loop or adaptive modulation. Kwon et al. evaluated a smart-mattress delivering closed-loop vibration stimulation (CLVS) coupled to autonomic cardiac rhythms. In individuals with poor sleep quality, CLVS reduced Wake after Sleep Onset (WASO) and increased both slow-wave activity and parasympathetic tone. These results illustrate how non-perceptible, ambient stimulation can meaningfully enhance sleep depth, while demonstrating the feasibility of fully embedded stimulation platforms suited for home environments.

In a complementary direction, Simons et al. tested short-duration transcranial electrical stimulation (tES) at 0.75 Hz in adults with sleep-onset insomnia. Their randomized crossover study found that slow-frequency stimulation reduced sleep-onset latency (SOL) by 53% compared with baseline and significantly outperformed a 25 Hz control frequency. The increase in frontal EEG coherence near the stimulation frequency highlights mechanistic alignment with the low-frequency processes described by Coon et al., underscoring that wearable tES may accelerate natural sleep onset by biasing cortical dynamics toward sleep-like rhythms.

Whereas, these stimulation studies sought to alter neurophysiology directly, Ypsilanti et al. introduce a different form of adaptive, real-time modulation through the “SleepCogni” active-feedback device. By integrating tactile cues, reaction-time monitoring, and physiological sensing, the device guides users into an “active rest” state aimed at reducing cognitive hyperarousal, one of the core maintaining factors in insomnia disorder. Their placebo-controlled trial demonstrated meaningful reductions in insomnia severity and improvements in sleep efficiency and total sleep time.

Beyond testing new sleep interventions and devices, this Topic also highlights innovations in sleep measurement and computational analysis that support the development of closed-loop systems. Liang et al. developed a convolutional neural network for automating sleep spindle detection across healthy and insomnia cohorts, achieving accuracy above 90%. Through transfer learning, spindle representations trained on healthy datasets generalized well to clinical sleep recordings, reducing reliance on large, labeled datasets—one of the major bottlenecks for real-time sleep staging and for closed-loop systems remarked upon by Coon et al.. Their findings underscore the importance of scalable, generalizable machine-learning models for enabling wearable devices to monitor brain states with sufficient precision for neurostimulation.

However, meta-analytic approaches highlight uncertainty across biofeedback studies, as illustrated by the systematic review and meta-analysis provided by, Recio-Rodriguez et al. evaluating neurofeedback interventions for insomnia. Curiously, across randomized controlled trials, “surface neurofeedback” (based on real time EEG data), showed an overall effect which favored improvements in sleep quality (PSQI scores) in control conditions over-active conditions, whereas insomnia symptom severity showed no significant change. This contribution is an important reminder that methodological rigor, including placebo controls and standardized protocol, remains essential. Recio-Rodriguez et al.'s conclusions resonate with the engineering constraints highlighted by Coon et al., emphasizing that device-based sleep interventions must be evaluated against strong control conditions and grounded in a clear understanding of the underlying physiological mechanisms.

Together, these contributions illustrate the breadth and momentum of the field. They highlight three converging trends:

1. Closed-loop, state-dependent stimulation is increasingly feasible and yields promising results

Whether through auditory, electrical, vibratory, or thermal pathways, interventions synchronized to endogenous physiology show promise for improving sleep continuity, deepening NREM sleep, and accelerating sleep onset.

2. An evolution toward multi-modal adaptive systems

This topic highlights a departure from devices that target one *sensory channel* (namely auditory stimulation) toward integrated multi-modal approaches such as skin-temperature feedback and tactile stimulation. Future work demonstrating a combination of approaches is warranted.

3. Open, transparent hardware and algorithms are essential for progress

Proprietary, “black box” sleep devices limit scientific replication and slow innovation. The field must move toward open-source hardware, transparent stimulation algorithms, and modifiable real-time decoding pipelines that researchers can inspect, validate, and refine. Such openness enables reproducibility, accelerates methodological improvements, and supports interoperable systems that can drive genuine advances in sleep neurotechnology.

## Conclusion

Taken together, the studies presented in this Research Topic demonstrate how neurotechnology, wearable systems, and computational methods are reshaping both the scientific understanding of sleep and the practical tools available to treat sleep disturbances. By integrating physiological precision, user-centered design, and open scientific frameworks, these approaches hold substantial promise for advancing sleep health across diverse populations, when applied correctly.

## References

[B1] Baron K. G. Hooker S. (2017). Next steps for patients who fail to respond to cognitive behavioral therapy for insomnia (CBT-I): the perspective from behavioral sleep medicine psychologists. Curr. Sleep Med. Rep. 3, 327–332. doi: 10.1007/s40675-017-0096-x

[B2] Grandner M. A. (2022). Sleep, health, and society. Sleep Med. Clin. 17, 117–139. doi: 10.1016/j.jsmc.2022.03.001 35659068

[B3] Koffel E. Bramoweth A. D. Ulmer C. S. (2018). Increasing access to and utilization of cognitive behavioral therapy for insomnia (CBT-I): a narrative review. J. Gen. Intern. Med. 33, 955–962. doi: 10.1007/s11606-018-4390-1 29619651 PMC5975165

[B4] Manber R. Alcántara C. Bei B. Morin C. M. van Straten A. A. (2023). Integrating technology to increase the reach of CBT-I: state of the science and challenges ahead. Sleep 46:zsac252. doi: 10.1093/sleep/zsac252 36308320

[B5] Perez-Pozuelo I. Zhai B. Palotti J. Mall R. Aupetit M. Garcia-Gomez J. M. . (2020). The future of sleep health: a data-driven revolution in sleep science and medicine. NPJ Dig. Med. 3:42. doi: 10.1038/s41746-020-0244-4 32219183 PMC7089984

[B6] Sateia M. J. Buysse D. J. Krystal A. D. Neubauer D. N. Heald J. L. (2017). Clinical practice guideline for the pharmacologic treatment of chronic insomnia in adults: an American Academy of Sleep Medicine clinical practice guideline. J. Clin. Sleep Med. 13, 307–349. doi: 10.5664/jcsm.6470 27998379 PMC5263087

